# Impact of arteriovenous fistula blood flow on serum il-6, cardiovascular events and death: An ambispective cohort analysis of 64 Chinese hemodialysis patients

**DOI:** 10.1371/journal.pone.0172490

**Published:** 2017-03-07

**Authors:** Zhizhi Hu, Fengmin Zhu, Nan Zhang, Chunxiu Zhang, Guangchang Pei, Pengge Wang, Juan Yang, Yujiao Guo, Meng Wang, Yuxi Wang, Qian Yang, Han Zhu, Wenhui Liao, Zhiguo Zhang, Ying Yao, Rui Zeng, Gang Xu

**Affiliations:** 1 Division of Nephrology, Tongji Hospital, Tongji Medical College, Huazhong University of Science and Technology, Wuhan, Hubei, China; 2 Department of Nephrology, Affiliated Hospital of Jining Medical University, Jining, Shandong, China; 3 Department of Geriatrics, Tongji Hospital, Tongji Medical College, Huazhong University of Science and Technology, Wuhan, Hubei, China; 4 School of Medicine and Health Management, Tongji Medical College, Huazhong University of Science and Technology, Wuhan, Hubei, China; The University of Tokyo, JAPAN

## Abstract

Flows (Qa) of arteriovenous fistula (AVF) impact the dialysis adequacy in hemodialysis (HD) patients. However, data for different access flow levels on outcomes related to long-term dialysis patients, especially in Chinese patients, are limited. Herein, we performed an ambispective, mono-centric cohort study investigating the association between the AVF flows and inflammation, cardiovascular events and deaths in Chinese hemodialysis patients bearing a radio-cephalic fistula (AVF) from 2009 to 2015. Twenty-three patients (35.9%) developed at least one episode of cardiovascular disease (CVD) in two years after AVF creation. AVF Qa, IL-6 and hsCRP were significantly higher in patients with CVD than in patients without CVD. Multi-factorial binary logistic regression analysis found that the independent and strongest risk factor for CVD in HD patients was serum IL-6, which showed a positive association with AVF Qa levels in patients. Therefore, the linkage between AVF Qa tertiles and adverse clinical outcomes (cardiovascular events and mortality) was examined over a median follow-up of five years. IL-6 was significantly increased in the high AVF Qa (>1027.13 ml/min) group. Patients with median AVF Qa showed the lowest morbidity and mortality of CVD according to the AVF Qa tertiles, whereas higher Qa was associated with a higher risk of CVD, and lower AVF Qa (600 ml/min ≤AVF Qa <821.12 ml/min) had a higher risk of non-CVD death. Therefore, keeping the AVF Qa at an optimal level (821.12 to 1027.13 ml/min) would benefit HD patients, improve long-term clinical outcomes and lower AVF-induced inflammation.

## Introduction

Arteriovenous fistula (AVF) surgery is the gold-standard procedure for vascular access to hemodialysis (HD) patients [1–2] and provides convenient and safe access for HD patients. Compared to prosthetic graft material, naive AVF is associated with increased patency rates, adequate blood flow and lower mortality rates [3] and is literally called the lifeline of long-term HD patients [4]. Satisfactory AVF flow (Qa) is necessary for dialysis adequacy. Low AVF Qa is generally indicative of access dysfunction, while high AVF Qa increases cardiac output (CO) and high-output cardiac failure [5].

However, Dr. Korsheed et al. reported in *Hemodialysis International* that patients with a high AVF Qa (>1000 mL/min) had a lower prevalence of LVH (Left ventricular hypertrophy) and a lower level of dialysis-induced cardiac injury [6], and another study by Lameire et al. published in *Nephrology Dialysis Transplantation* showed hemodialysis does not impair ventricular function after AVF surgery over 2 years, which conveys the controversy on AVF flow levels and the initiation or aggravation of cardiac injury [7].

Although hemodialysis therapy for end-stage chronic renal failure has been used for almost forty years, the mortality rate of hemodialysis patients remains unacceptably high, and cardiovascular disease (CVD) causes more than 50% of the deaths among long-term hemodialysis patients [8–9]. A recent Chinese study reported that 52.9% of early deaths within the first hemodialysis year were due to cardiovascular and cerebrovascular diseases [10]. Therefore, defining the pathogenesis of CVD in hemodialysis patients, especially clarifying the relationship between AVF access and cardiac injury in Chinese hemodialysis patients, is paramount.

Arteriovenous fistulas not only develop anatomical malformations but also induce local endothelium dysfunction, vascular wall proliferation, and the release of endothelial-related inflammatory cytokines [11]. Inflammatory indicators were shown to have predictive value for mortality in HD patients [12]. Recent studies have shown that long term chronic inflammation plays a crucial role in the development of CVD, and inflammatory cytokines IL-6 and C-response protein (CRP) have been increasingly recognized as most important [13–16]. Madhumathi Rao [15] et al. reported in *American Journal of Kidney Diseases* that plasma IL-6 levels are strongly associated with hemodialysis comorbidity and are a powerful predictor of cardiovascular and all-cause mortality. IL-6 is a type of endothelial cell cytokine that is secreted in response to mechanical stimuli and leads to an accelerated inflammatory response [17]; however, whether IL-6 is associated with arteriovenous fistula–induced inflammation remains unknown [13–14].

In this study, we report the results of an ambispective cohort study on stable long-term HD patients after a follow-up period of five years. We hypothesized that AVF blood flow plays an important role on inflammatory status, explored the relationship between AVF Qa and serum IL-6 levels, and investigated the cardiovascular disease morbidity and mortality of HD patients according to the AVF Qa tertiles in five-year follow-up.

## Material and methods

### Patients

This study complied with the Declaration of Helsinki. A total of 79 dialysis patients were recruited in the Blood Purification Center of Tongji Hospital from June 2009 to March 2010. All patients had radio-cephalic AVF opened by specialists with at least 2 years of specialized experience. As criteria for inclusion in the study, patients who had an AVF opened for the first time and with at least 2 years of AVF use were included. Patients with cardiac, hepatic disease or malignancy, active inflammation (e.g.: tuberculosis, septicemia), use of immunosuppressive agents or blood products in the past 3 months, a scheduled or recently (6 months) failed transplant, or a scheduled change of dialysis mode were excluded. Four patients with an AVF Qa less than 600 ml/min were excluded, according to the requirements of the NKF-K/DOQI Clinical Practice Guidelines for Vascular Access. During follow-up, 11 patients having a variation percentage of AVF Qa more than 3% were excluded. A total of 64 patients completed follow-up for five years.

All patients were subjected to regular hemodialysis procedures, receiving standard bicarbonate hemodialysis 3 times per week for 4 hours each time, with biocompatible hollow-fiber dialyzers at a dialysate flow rate of 500 mL/min and a blood flow rate of 200~300 mL/min (Rexeed-L, Asahi, Tokyo, Japan), which was disposable. All the patients signed informed consent to participate in the study, which was approved by the Institutional Ethics Committee on Research Ethics of the Huazhong University of Science and Technology,Tongji Hospital.

We retrospectively recorded cardiovascular events during the two years prior to patient enrollment. Cardiovascular events were defined as angina pectoris, myocardial infarction, and congestive heart failure. Patients were classified into two groups according to the two-year retrospective data. Twenty- three patients with at least one episode of cardiovascular events were in the CVD group, and 41 patients with no history of cardiovascular events were in the non-CVD group.

### Clinical data

Demographic, medical, and socioeconomic information were inquired during the baseline phase of the study. Baseline predialysis blood samples were obtained before AVF Qa monitoring. Blood calcium, hemoglobin, albumin and hsCRP were measured by standard laboratory techniques with an automatic analyzer. All the analyses were performed on fresh blood samples.

### AVF Qa and CO monitoring

AVF Qa and CO was evaluated using an ultrasound dilution Transonic Hemodialysis Monitor HD02 (Transonic Systems, Inc., Ithaca, NY, USA). Measurements were performed by experienced specialists between the first half hour and the last hour of every dialysis session. AVF Qa and CO were determined by the average of three separate measurements taken approximately 5–10 min apart. AVF Qa and CO were monitored every 6 months by specialists.

### Measurement of inflammatory cytokines

Blood was immediately placed on ice and centrifuged at 3000 rpm for 5 minutes within one hour of sample collection. Plasma was aliquoted and stored at -80°C, avoiding repeated freeze-thaw cycles. Interleukin-2 (IL-2), Interleukin-6 (IL-6), Interleukin-10 (IL-10) and tumor necrosis factor (TNF-α) were measured by Cytometric Bead Array (BD^™^). The samples were thawed before measurement, specified reagents were added into samples according to the instruction manual, data were acquired on a flow cytometer and analyzed using FCAP Array Software provided by the company.

### Measurement of lymphocyte immunophenotyping

Each blood sample was aliquoted into three tubes, and the following antibodies (BD, USA) were added to each cell tube: CD3-FITC, CD8-PE, CD45-PerCP and CD4-APC; CD45-PerCP, CD3-FITC, CD16-PE, CD56-APC; CD19-PE. Erythrocytes were lysed using BD FACS lysing solution. Cells were pelleted by centrifugation (1000~1500 rpm for five minutes at room temperature), the supernatant was aspirated and the cells were washed three times in phosphate-buffered saline (PBS). After the final wash, cells were resuspended in 300 μl PBS. At least 30,000 events were analyzed in a BD FACScalibur flow cytometer. Analysis was performed using CD45 vs. orthogonal light scatter (SSC) gating. Acquisition and analysis was made using Cellquest software (BD).

### Follow-up

After the cross-sectional study, the patients were followed up for five years from March 2010 to May 2015, depending on the time of randomization. In our study, the patients followed up were treated with maintenance hemodialysis; they came to hospital for hemodialysis three times every week. The investigation was done during this time. We recorded their cardiovascular events and deaths. In 2015, after the five-year follow-up, the patients were classified into the following three groups, according to the AVF Qa tertiles: 600 ml/min ≤AVF Qa <821.12 ml/min was the low AVF Qa group; 821.12 ml/min≤AVF Qa≤1027.13 ml/min was the median AVF Qa group; AVF Qa>1027.13 ml/min was the high AVF Qa group. HD patients were required to report their condition to the specialists during every dialysis. The times of cardiovascular events and deaths were recorded.

### Statistical analysis

Analysis was performed using SPSS software version 18.0(SPSS, Inc., Chicago, IL). *P* values of less than 0.05 were considered significant. Data were expressed as medians and quartiles for continuous variables. Categorical data were expressed as proportions. Comparisons between two groups were performed using Mann-Whitney^’^s U-test. Chi-square statistics were performed for categorical data. Explanatory variables used Spearman rank analysis, nominal variables used the point-biserial correlation analysis, and linear regression analyses were applied to evaluate the correlation between all available data. Binary logistic regression analysis was performed on the risk factors of CVD according to comparisons between the two groups.

## Results

### Surveillance of arteriovenous fistula

AVF Qa and cardiac output (CO) were measured by means of the ultrasound dilution Transonic Hemodialysis Monitor HD02 on all patients, and four patients were found to have AVF Qa less than 600 ml/min. On the basis of the NKF-K/DOQI Clinical Practice Guidelines for Vascular Access [11], patients with AVF Qa less than 600 ml/min are already at high risk for subsequent dysfunction, and they were excluded in analysis. During the follow-up, AVF Qa was monitored every half a year by specialists, and seven patients were found to have a variation percentage of AVF Qa more than 3% due to arteriovenous fistula stenosis or thrombosis. These patients were excluded. Finally, a total of 64 patients were followed-up for five years, and their initiated AVF Qa ranged from 620 ml/min to 1490 ml/min and the mean AVF Qa was 954.5 ml/min.

### Risk factors of CVD in hemodialysis patients

Of the 64 patients, there were 44 males and 20 females. The primary diseases of ESRD consisted of chronic glomerulonephritis in 30 patients (46.8%), lupus nephritis in two (3.12%), hypertensive renal disease in seven (10.9%), gouty nephropathy in two (3.12%), diabetic nephropathy in 10 (15.6%), and uncategorized in 13 (20.3%) (Table 1). From the past two years to the date of enrollment, there were 23 HD patients who developed at least one episode of a cardiovascular event during the retrospective period, and 41 patients without history of cardiovascular events formed the non-CVD group. After five-years follow-up, the CVD morbidity and mortality is different between CVD group and non-CVD group (S3A and S3B Fig). The majority death caused by heart failure or arrhythmia.13 patients died in the CVD group and 2 died in non-CVD group in the following five years,and 11 patients developed new CV event in the non-CVD group. The age, AVF Qa, IL-6 and hsCRP in patients with CVD were significantly higher than in patients without CVD {69.0(63.5~71.0) vs. 53.5(44.0~58.0) year, *P*<0.05; (1120±192) vs. (893±189) ml/min, *P*<0.05; 4.86(2.96~7.85) vs. 2.20(1.80~3.10) pg/ml, P<0.01; 11.75(3.83~31.53) vs. 4.45(1.05~6.68) mg/L, *P*<0.05}. There were no differences in peripheral blood T cells, B cells, Th cells, Ts cells and NK cells (Table 1). Therefore, age, AVF Qa, IL-6 and hsCRP were recognized as risk factors of CVD (Table 1).

**Table 1:** Demographic and clinical characteristics of the 64 HD patients subdivided by the presence or absence of Cardiovascular Disease (CVD) after two years of hemodialysis, before the five-years follow-up.

Characteristics	Patients with CVD	Patients without CVD	P value
Number	23	41	
Median age (Y)	68.0(44~74.0)	54.0(44.0~61.5)	0.01
Sex ratio (M/F)	18/5	26/15	0.73
Duration of HD (M)	30.0(5.5~52.3)	25.0(10.5~50.0)	0.89
Location of fistula (Radial/ Brachial)	22/1	40/1	0.42
BMI (kg/m^2^)	20.69±2.49	22.04±3.43	0.30
EPO (U/week)	0.80(0.40~1.00)*10^4^	0.80(0.40~1.00)*10^4^	0.93
Causes of ESRD			0.42
Chronic glomerulonephritis	12	18	
Hypertension	2	5	
Gout	0	2	
SLE	0	2	
DM	6	4	
Unknown	3	10	
Antihypertensive therapy			0.56
Calcium antagonist	6	21	
ACEI or ARB	7	8	
ß Blocker	11	20	
CO (L/min)	6.07±1.51	6.38±1.77	0.78
AVF Qa (ml/min)	1105±176	918±215	0.01
Ca (mmol/L)	2.17±0.33	2.27±0.26	0.63
P (mmol/L)	1.02±0.21	2.05±0.62	0.08
Hb (g/L)	99.7±13.6	101.9±15.6	0.52
ALB (g/L)	38.1±3.5	38.2±6.5	0.66
iPTH (ng/L)	589.35±412.43	606.72±612.86	0.73
HD/HDF	11/12	10/31	0.28
Anticoagulant (UFH/LMWH)	10/13	16/25	0.43
KT/V	1.68±0.19	1.64±0.19	0.35
HsCRP(mg/L)	8.75()3.83~18.40)	3.80(1.00~7.20)	0.03
IL-2 (pg/ml)	2.73(2.19~6.22)	2.59(2.01~4.50)	0.41
IL-6 (pg/ml)	6.00(4.40~11.49)	2.60(1.96~4.73)	0.002
IL-10 (pg/ml)	1.85(1.42~3.36)	1.52(1.12~2.37)	0.13
TNF-α (pg/ml)	1.60(1.21~2.92)	1.51(1.14~2.18)	0.42
T cells	69.00(63.00~78.75)	74.00(64.25~81.75)	0.58
Th cells	42.00(35.25~52.50)	41.50(37.00~49.00)	0.90
Ts cells	24.00(22.25~26.50)	24.00(20.75~32.75)	0.95
Th/Ts	1.67(1.51~2.15)	1.58(1.37~2.09)	0.59
B cells	6.50(3.00~16.00)	7.50(5.75~11.25)	0.42
NK cells	15.50(7.75~23.25)	12.50(6.00~22.00)	0.81

Data are expressed as medians and quartiles for continuous variables that were not normally distributed and as the means and standard deviations for variables that were normally distributed. Comparisons between groups were performed by chi-squared tests or nonparametric tests. SLE, Systemic Lupus Erythematosus; DM, diabetic mellitus; EPO, Erythropoietin; BMI, body mass index; ACE, angiotensin converting enzyme; ARB, angiotensin II receptor blocker. P<0.05 was considered statistically significant.

To determine the relationship between serum IL-6 and AVF Qa and cardiovascular outcomes in the HD population, patients were classified into IL-6 low and high groups by the IL-6 median level at 3.15 pg/ml. Patients in the high IL-6 group had a higher level of AVF Qa (835.40±159.01 vs. 1069.00±210.62, *P*<0.001) and CVD morbidity (54.17% vs. 78.26%, *P*<0.05) compared to the low IL-6 group (S1 Fig and S1 Table). These data indicated that the IL-6 level is associated with CVD morbidity of HD patients, and it is also related to the levels of AVF Qa.

### Multi-factorial binary logistic regression analysis of CVD risk factors in hemodialysis patients

In our study, of four inflammatory cytokines, only IL-6 showed a significant difference between the CVD group and the non CVD group, consistent with previous reports that IL-6 is a stronger predictor of total and cardiovascular mortality [18, 19]. The efficacy of IL-6 and hsCRP in predicting CVD was assessed by means of receiver operating characteristic (ROC) curve analysis (S2 Fig). The ROC curve analysis showed the Area Under Curve (AUC) and the 95% confidence interval (CI) of IL-6 and hsCRP were {0.875, (0.707~1.000), *P*<0.01; 0.771, (0.553~0.989), *P*<0.05}, respectively; X^2^ statistics were performed on the predictive values of IL-6 and hsCRP and showed no difference, indicating that IL-6 was equivalent to HsCRP in predicting CVD. As shown in Table 1, the age, AVF Qa, IL-6 and hsCRP in patients with CVD were significantly higher than in patients without CVD. Considering the powerful predictive value of IL-6, hsCRP was excluded from binary logistic regression analysis, and three other risk factors were input into the regression, namely age, AVF Qa and IL-6. Finally, IL-6 was output as the independent risk factor of CVD {Hazard ratio = 1.456, β = 0.376, *P* = 0.014, 95% CI (1.078~1.967)} (Table 2). Age and AVF Qa did not enter into the equation. These data suggested that IL-6 predicts the risk of CVD in HD patients.

**Table 2:** Multi-factorial binary logistic regression analysis of CVD risk factors in the 64 HD patients after two years of hemodialysis, before the five-years follow-up.

Variable	B(β)	HR	P	95% CI
IL-6	0.376	1.456	0.014	1.078~1.967

Nb: a Method: Forward: Conditional

b constant is included in the model

c Initial -2 Log Likelihood: 49.589

d Estimation terminated at iteration number 5 because parameter estimates changed by less than 0.001.

*B* means partial regression coefficient, age, Qa and HsCRP did not enter into equation.

### Association of AVF Qa and IL-6

**Fig 1:**
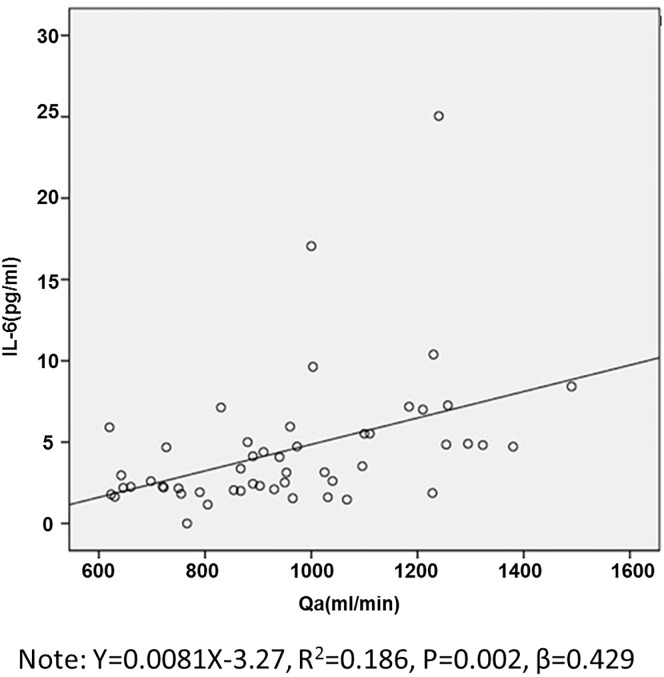
Linear relationship between Qa and IL-6 in the 64 HD patients before the five-years follow-up.

In Spearman rank analysis, IL-6 showed a positive correlation with AVF Qa and hsCRP, respectively among fourteen variables (Table 3). In consideration of the strong correlation of hsCRP and IL-6, hsCRP was excluded from the linear regression analysis to avoid co-linearity. Spearman rank analysis was performed between AVF Qa and the other fourteen variables, showing a positive correlation between AVF Qa and IL-6 (Table 3). The linear regression analysis showed that AVF Qa was independently related to IL-6, and a linear equation model showed AVF Qa is positively correlated with IL-6 in Fig 1 (Y = 0.0081X-3.27 R^2^ = 0.186, P = 0.002, β = 0.429).

**Table 3:** Spearman rank and point-biserial correlation analysis between IL-6, AVF Qa and thirteen other variables in the 64 HD patients after two years of hemodialysis, before the five-years follow-up.

Variable	DM	Sex	Duration of HD	Age	ACEI/ARB	Ca	AVF Qa	IL-6	CO	Hb	ALB	hsCRP	EPO	BMI	iPTH
IL-6	0.128	0.245	-0.095	0.270	-0.229	0.071	0.511**		0.051	0.192	-0.137	0.452***	-0.30	0.032	-0.35
AVF Qa	0.091	0.16	0.153	0.237	0.167	-0.028		0.511**	0.272	-0.014	-0.13	0.199	-0.192	0.092	-0.40

R: correlation coefficients,

** *P*<0.01,

*** *P*<0.001,

P<0.05 was considered statistically significant.

### Impact of AVF Qa on risk of cardiovascular events and death

Because serum IL-6 was positive correlated with AVF Qa, we followed the patients for five years to explore the optimal AVF Qa levels for HD patients. The patients were classified into the following three groups according the AVF Qa tertiles: a low group with AVF Qa in 600 ml/min≤ AVF Qa <821.12 ml/min, a median group with AVF Qa in 821.12 ml/min≤AVF Qa≤1027.13 ml/min, and AVF Qa>1027.13 ml/min as a high AVF Qa group.

**Fig 2:**
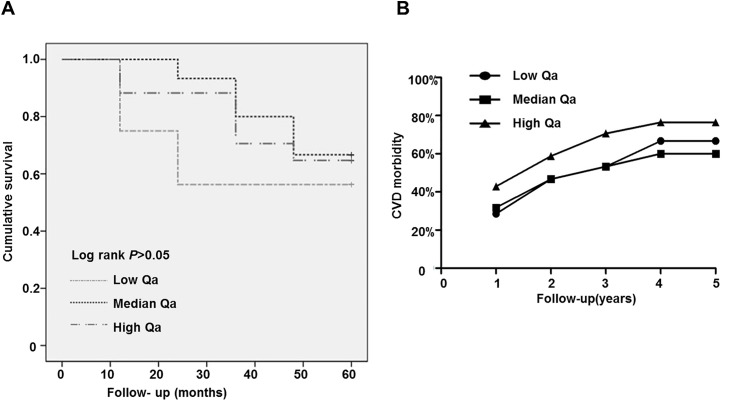
Mortality and CVD morbidity of the 64 HD patients in different AVF Qa groups after the five-years follow-up.

We found that serum IL-6 and Kt/V are significantly increased in both the median and high AVF Qa groups; however, TNF-α and IL-2 are only increased in the high AVF Qa group, and there are no differences in T cells, B cells, Th cells, Ts cells and NK cells (Table 4). Age, gender and IL-10 showed no differences among the groups (Table 4). After five years of follow-up, patients in the median AVF Qa group showed the lowest mortality (Fig 2A) compared to the low and high AVF Qa patients. Patients in the median AVF Qa group also showed lower morbidity of CVD, similar to the low AVF Qa group (Fig 2B, Table 4), compared to patients in the high AVF Qa group. These data indicated that AVF Qa induces IL-6 related inflammation, which consequently causes cardiovascular events, whereas low AVF Qa increases the all-cause death of patients via non-IL-6 mechanisms. The AVF Qa at the median level (821.12 to 1027.13 ml/min) benefits long-term HD patients.

**Table 4:** Clinical characteristics of inflammatory cytokines and immune cells, in which the 64 HD patients were subdivided into different AVF Qa groups before the five-years follow-up.

Variable	Low AVF Qa	Median AVF Qa	High AVF Qa	P1 value (L vs. M)	P2 value (L vs. H)	P3 value (M vs. H)
Number	21	22	21			
Age (year)	50.0(40.0~58.0)	56.0(50.0~70.0)	61.0(38.0~71.0)	0.12	0.25	0.65
Sex ratio (M/F)	10/11	12/10	12/9	0.95 r e 74s	0.14	0.36
AVF Qa (ml/min)	703.6±63.0	925.8±55.0	1207.9±125.0	0.0001	0.0001	0.001
KT/V	1.56±0.18	1.67±0.13	1.73±0.29	0.055	0.047	0.36
IL-2 (pg/ml)	2.57(1.78~3.55)	2.22(2.03~2.74)	3.89(2.61~5.99)	0.78	0.02	0.02
IL-6 (pg/ml)	2.19(1.79~2.60)	3.37(2.31~5.00)	4.90(3.07~7.22)	0.01	0.002	0.14
IL-10 (pg/ml)	1.55(1.02~2.47)	1.52(1.18~2.53)	1.88(1.29~3.01)	0.98	0.53	0.37
TNF-α (pg/ml)	1.45(1.19~1.56)	1.46(1.01~2.21)	1.95(1.36~2.57)	0.64	0.04	0.18
T cells	75.00(65.50~81.50)	74.50(67.00~78.25)	79.00(71.00~83.00)	0.74	0.40	0.23
Th cells	42.00(34.00~54.00)	48.00(39.5~52.00)	48.00(40.00~51.50)	0.79	0.62	0.88
Ts cells	22.00(20.00~28.50)	24.50(23.00~26.75)	27.00(24.00~35.00)	0.25	0.10	0.45
Th/Ts	1.77(1.35~2.69)	1.83(1.64~2.17)	1.52(1.29~2.08)	0.95	0.49	0.46
B cells	7.00(4.50~11.50)	10.50(7.25~14.50)	13.00(9.50~13.00)	0.50	0.08	0.45
NK cells	11.00(6.00~25.00)	11.50(5.75~22.50)	6.00(4.00~13.50)	0.84	0.18	0.45

Data are expressed as medians and quartiles for continuous variables that were not normally distributed or as the means and standard deviations for variables that are normally distributed. Comparisons between groups were performed by chi-squared tests or nonparametric tests. P<0.05 was considered statistically significant. L means low AVF Qa, M means median AVF Qa, H means high AVF Qa.

## Discussion

In this ambispective cohort study, our results corroborated the findings of previous studies [15] that IL-6 was an independent and strong risk factor of CVD in hemodialysis patients. We observed that the serum IL-6 level was positively correlated with arteriovenous fistula (AVF) flow. We further investigated the association of AVF flow (Qa) with cardiovascular events and deaths during the following 5-year interval. We found that Chinese hemodialysis patients with AVF Qa in the median range (821.12 to 1027.13 ml/min) showed the lowest morbidity and mortality of CVD in five-year follow-up compared to low and high AVF Qa patients, suggesting the optimal initiation of AVF Qa of HD patients will benefit patients with improved long-term clinical outcomes.

Cardiovascular disease (CVD) is the leading cause of death among hemodialysis patients [8–9]. Increasing evidence proves that inflammation is the most powerful risk factor of CVD in these patients, strongly predicting all-cause and cardiovascular mortality [20, 21]. Furthermore, inflammation predicts the development of CVD in healthy people [22]. Inflammation plays an important role in the development of atherosclerosis, which is an important risk factor of CVD [13–14, 23].

In our study, AVF Qa, IL-6 and hsCRP were significantly higher in patients with CVD than without CVD. HsCRP was shown to have predictive value for mortality in HD patients, although it is thought to be downstream from IL-6 [14–15, 24–26, 27–28]. IL-6 is a pleiotropic and predominant cytokine involved in many physiological and pathological processes and is also a mediator and marker of the chronic inflammatory response in HD patients [15, 23–26]. IL-6 is also considered a stronger predictor of outcomes when compared to hsCRP in HD patients [18]. The reasons for this difference are as follows: first, being located upstream in the cascade of events that lead to the synthesis of many acute-phase reactants, IL-6 is the best marker for inflammatory burden. Second, IL-6 varied less than hsCRP, leading to a more accurate evaluation. Third, the toxic effects of IL-6 on the heart and peripheral vasculature are stronger than the effects of hsCRP [19]. Considering the powerful predictive value of IL-6, hsCRP was excluded from binary logistic regression analysis. In this study, age, AVF Qa and IL-6 were accepted into the model of binary logistic regression, but only IL-6 was finally identified as an independent risk factor of CVD {Hazard ratio = 1.456, β = 0.376, *P* = 0.014, 95% CI (1.078~1.967)} (Table 2).

We further demonstrated that patients in the high IL-6 group had higher levels of AVF Qa and CVD morbidity compared to the low IL-6 group, indicating that IL-6 was associated with AVF Qa and CVD. We analyzed the relationship between AVF Qa and IL-6. We found that AVF Qa is positively correlated with IL-6, suggesting that AVF flow itself contributes to increased inflammatory cytokine levels in hemodialysis patients. How does high AVF Qa promote the production of IL-6? We predicted that a possible reason is that constant and high access flow imposes strong shear stress on vascular endothelial cells and cause injury, which then induces the occurrence and development of vascular inflammation [29]. Further, we analyzed the lymphocyte subset in different IL-6 groups, and there were no differences between the two groups, suggesting that, the other IL-6 increasing reason may be the strong shear stress induces the IL-6 secretion in peripheral blood cells, but not increasing their cell numbers.

As a result, patients displayed greater inflammation as determined by high IL-2, IL-6 and TNF-α levels in the high AVF Qa group compared with patients in the low AVF Qa group. As IL-6 related inflammation causes cardiovascular events, after five-year follow-up, patients in the high AVF Qa group showed a higher morbidity of CVD compared with patients in the low AVF Qa group. However, it is interesting that patients in the low AVF Qa group had higher mortality and lower morbidity of CVD compared with the high AVF Qa group. We found that, although above normal level, the Kt/V in the low Qa groups was lower than the high Qa group (Table 4). So we predicted that low AVF Qa access flows may cause relatively insufficient hemodialysis in these patients. This evidence indicated that AVF Qa predicts CVD morbidity and mortality, and the optimal initiated AVF Qa would benefit HD patients with improved long-term clinical outcomes.

These findings allow us to consider that anti-inflammation therapy could reduce AVF related morbidity and mortality. As the AVF Qa, to a certain extent, is associated with peripheral blood IL-6 production and the morbidity of CVD in hemodialysis patients, keeping AVF Qa in an appropriate range or the use of anti-IL-6 monoclonal antibodies are likely to alleviate an inflammatory status and benefit hemodialysis patients. We recommend regular AVF flow monitoring and the AVF flow should be restricted to an optimal range to achieve long-term clinical outcomes and benefits in hemodialysis patients.

## Supporting information

S1 Fig CVD morbidity of HD patients in different IL-6 groups. (TIF)

S2 Fig Receiver Operating Characteristic (ROC) curve analysis of the efficacy of IL-6 and hsCRP in predicting CVD. (TIF)

S3 Fig The CVD morbidity and mortality in different CVD group after five-year follow up. (TIF)

S1 Table CVD morbidity of HD patients in different IL-6 groups. (DOCX)
