# Supplementary figures and images for: Impact of arteriovenous fistula blood flow on serum il-6, cardiovascular events and death: An ambispective cohort analysis of 64 Chinese hemodialysis patients
Source: PLoS One. 2017 Mar 7;12(3):e0172490. doi: 10.1371/journal.pone.0172490 (PMC5340356; doi:10.1371/journal.pone.0172490)

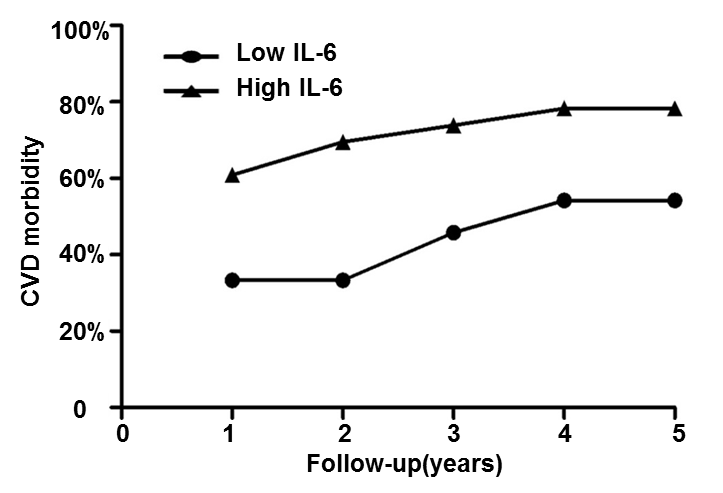

Supplement: S1 Fig — (TIF) [file pone.0172490.s001.tif]

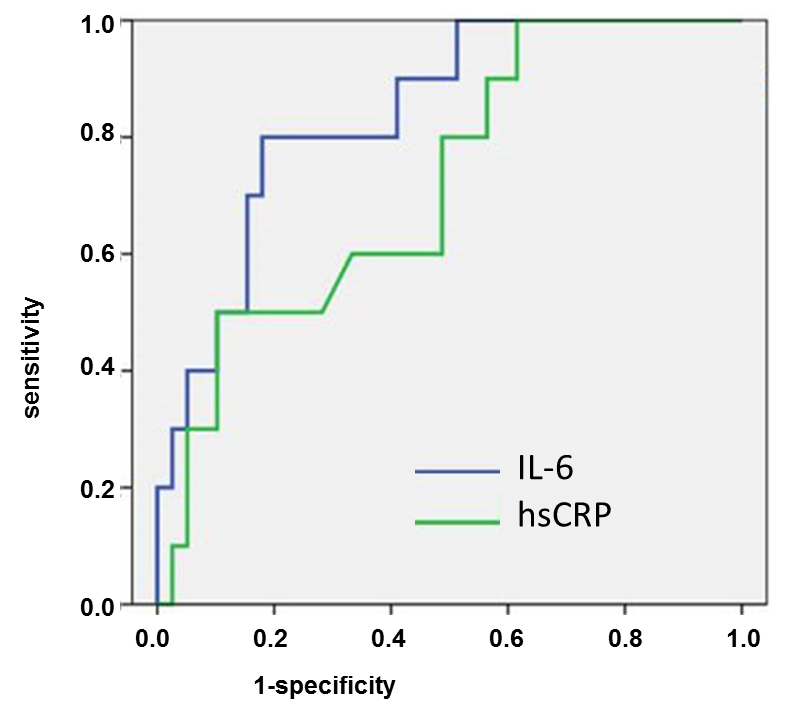

Supplement: S2 Fig — (TIF) [file pone.0172490.s002.tif]

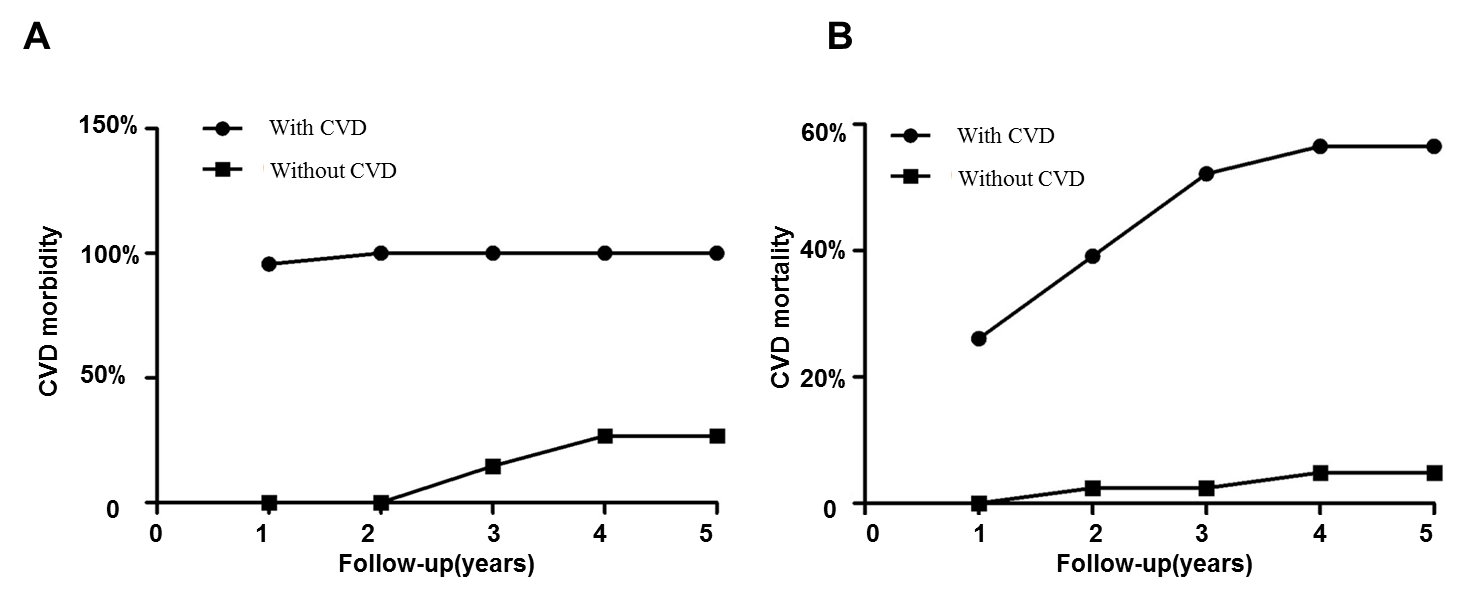

Supplement: S3 Fig — (TIF) [file pone.0172490.s003.tif]
