# Supplementary material for: Impact of arteriovenous fistula blood flow on serum il-6, cardiovascular events and death: An ambispective cohort analysis of 64 Chinese hemodialysis patients
Source: PLoS One. 2017 Mar 7;12(3):e0172490. doi: 10.1371/journal.pone.0172490 (PMC5340356; doi:10.1371/journal.pone.0172490)
Supplement: S1 Table — (DOCX) [file pone.0172490.s004.docx]

**S1 Table** CVD morbidity of HD patients in different IL-6 groups

| Variable | Low IL-6 | High IL-6 | P value |
| --- | --- | --- | --- |
| AVF Qa (ml/min) | 835.40±159.01 | 1069.00±210.62 | 0.0001 |
| CVD morbidity* | 54.17% | 78.26% | 0.03 |

Patients were classified into IL-6 low and high groups by the IL-6 median level at 3.15 pg/ml. *these data are follow-up five-year data.
